# Terbinafine Resistance in *Trichophyton rubrum* and *Trichophyton indotineae*: A Literature Review

**DOI:** 10.3390/antibiotics14050472

**Published:** 2025-05-07

**Authors:** Aditya K. Gupta, Susmita, Hien C. Nguyen, Amanda Liddy, Vasiliki Economopoulos, Tong Wang

**Affiliations:** 1 Division of Dermatology, Department of Medicine, Temerty Faculty of Medicine, University of Toronto, Toronto, ON M5S 3H2, Canada; 2 Mediprobe Research Inc., London, ON N5X 2P1, Canada; ssusmita@mediproberesearch.com (S.); hnguyen@mediproberesearch.com (H.C.N.); aliddy@mediproberesearch.com (A.L.); veconomopoulos@mediproberesearch.com (V.E.); twang@mediproberesearch.com (T.W.)

**Keywords:** terbinafine, squalene epoxidase, *Trichophyton rubrum*, *Trichophyton indotineae*, antifungal drug resistance

## Abstract

Background/Objectives: Terbinafine has been the gold standard for the management of superficial fungal infections. The etiological agent generally is *Trichophyton rubrum* (*T. rubrum*); however, there has been increased reporting of a new terbinafine-resistant strain of the *T. mentagrophytes complex* (*T. mentagrophytes* ITS genotype VIII otherwise known as *T. indotineae*). Here, we review the epidemiology, clinical features, diagnosis, and treatment of *T. rubrum* and *T. indotineae* infections. Methods: We conducted a systematic literature search using PubMed, Embase (Ovid), and Web of Science, resulting in 83 qualified studies with data summarized for clinical features, antifungal susceptibility, and terbinafine resistance mechanisms and mutations. Results: Dermatophytosis is most commonly caused by *T. rubrum*; however, in certain parts of the world, especially in the Indian subcontinent, *T. indotineae* infections have been reported more frequently. The majority of *T. rubrum* isolates remain susceptible to terbinafine (over 60% of isolates show MIC_50_ and MIC_90_ < 0.5 µg/mL). In contrast, for *T. indotineae*, 30% of isolates exhibit MIC_50_ ≥ 0.5 µg/mL and 80% exhibit MIC_90_ ≥ 0.5 µg/mL. Frequently detected squalene epoxidase (*SQLE*) mutations in *T. rubrum* are Phe397Leu/Ile (41.6%) and Leu393Phe (20.8%); in *T. indotineae*, these include Phe397Leu (33.0%) and Ala448Thr (24.5%). Other potential terbinafine resistance mechanisms in *T. rubrum* and *T. indotineae* are discussed. Conclusions: *T. rubrum* generally remain susceptible in vitro to terbinafine in contrast to *T. indotineae*. The essential components of an effective antifungal stewardship emphasize accurate clinical and laboratory diagnosis, susceptibility testing, and appropriate antifungal therapy selection with a multidisciplinary approach.

## 1. Introduction

Due to high infection rates, the global cost of treating dermatophytes is approximately USD 500 million each year [1]. In particular, it is estimated that in the United States approximately 22.4 million antifungal prescriptions are filled annually, which is enough to provide one prescription for every 15 people [2]. Additionally, reports have shown that dermatophyte infections represent a major portion of outpatient consultations for fungal diseases, with a notable prevalence of self-diagnosis (61.4%) and self-treatment (55.5%) among patients [2,3].

Terbinafine was first approved by the US FDA in 1992 and was the gold standard antifungal therapy [4,5,6]. Initially, terbinafine resistance was rarely reported, with the first confirmed case documented in the early 2000s [7,8,9]. The past decade has seen a significant rise in terbinafine resistance, coinciding with an increase in treatment failures and infection relapses [9,10].

Antifungal resistance can drive higher infection rates and contribute to the global spread of fungal diseases [11]. Clinical terbinafine-resistant dermatophyte isolates, specifically among anthropophilic clonal “offshoots” of *Trichophyton mentagrophytes* (*Trichophyton indotineae*), had an epicenter in the Indian subcontinent and have since been documented across Europe, Asia, the Americas, Australia, and Africa [12,13,14,15,16,17]. Infections acquired outside of South Asia have also been reported recently, reflecting an evolving outbreak scenario [18].

## 2. Clinical Features

*Trichophyton rubrum* (*T. rubrum*), the most prevalent dermatophyte and the leading cause of dermatophytosis globally [19], was also recently associated with emerging terbinafine resistance [20]. *T. indotineae*, originating in South Asia and spreading worldwide, with reported cases in Europe, the United States, and Canada, was recently identified with documented resistance to antifungals [21].

*T. rubrum* may manifest with dry, annular scaly plaques, affecting the feet (tinea pedis), corporis, cruris, and nails (onychomycosis), with relatively mild and slowly progressing lesions [22,23]. *T. indotineae* affects primarily the groin (tinea cruris), body (tinea corporis), and face (tinea faciei). *T. indotineae* can result in lesions that are intensely pruritic with a burning sensation and a high degree of inflammation. Other presentations include erythematous plaques and lesions with multiple concentric rings [21]. The eruption may become chronic and recalcitrant to treatment. Both species are contagious via human-to-human transmission; notably, there has been an epidemiological shift in the dominant dermatophyte species in India, with *T. indotineae* surpassing *T. rubrum* in prevalence (Table 1) [24].

## 3. Terbinafine Susceptibility

### 3.1. Literature Search

**Figure 1:**
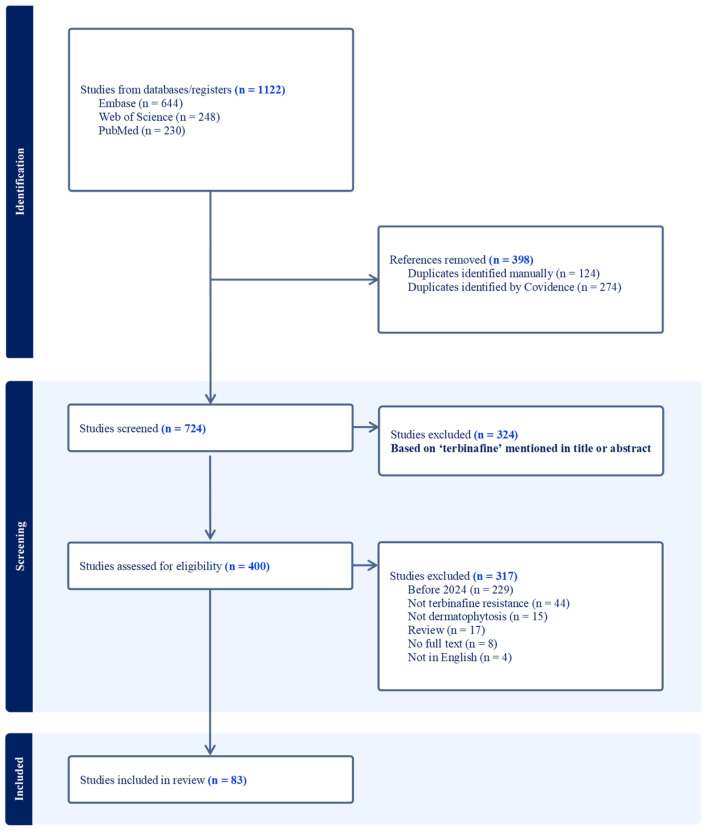
PRISMA flow diagram illustrating the selection process for studies included in the review. Literature searches were performed in PubMed, Embase (Ovid), and Web of Science. The retrieved records (n = 1122) were deduplicated with 398 duplicates removed (124 manually and 274 by Covidence). Then, 641 studies were filtered through critical inclusion criteria, resulting in 83 studies selected.

A systematic literature search (Figure 1) was conducted in January 2025 to identify and analyze recent investigations (2024–present) of in vitro terbinafine resistance in *T. rubrum* and *T. indotineae*. Literature searches were performed in three electronic databases: PubMed, Embase (Ovid), and Web of Science, the keywords and subject headings included “dermatophyte”, “*Trichophyton*”, and “terbinafine”. All retrieved records (n = 1122) were imported into Covidence for deduplication and screening. Reviews, non-English articles, and articles without full-text availability were excluded. Data extraction, conducted independently by authors A.L., S., and H.N., included study characteristics (authors, publication year, location), patient demographics, terbinafine susceptibility, resistance mechanisms, and treatment regimens, with discrepancies resolved through discussion with author A.K.G.

### 3.2. In Vitro Terbinafine Susceptibility

**Figure 2:**
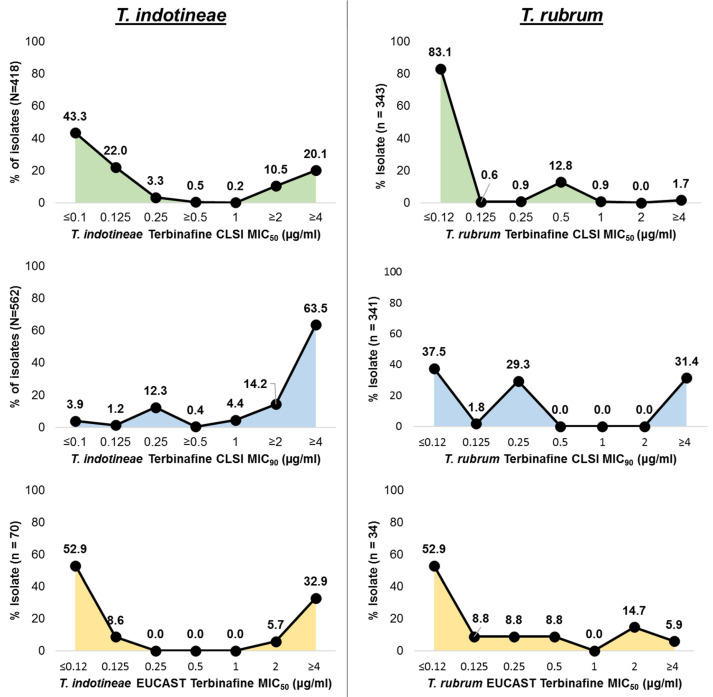
Terbinafine susceptibility patterns of *T. indotineae* and *T. rubrum* [25,26,27,28,29,30,31,32,33,34,35,36,37,38,39,40,41,42,43,44,45,46,47,48,49,50,51,52,53,54,55,56,57,58,59,60,61,62,63,64,65,66,67,68,69]. Terbinafine resistance studies reporting MIC values for 50% (MIC_50_) and 90% (MIC_90_) inhibition of isolate growth, respectively, under CLSI (green: MIC_50_; blue: MIC_90_) and EUCAST protocols (yellow: MIC_50_).

Antifungal susceptibility testing (AFST) can provide insights into the terbinafine resistance profiles of *T. rubrum* and *T. indotineae*, thereby helping to guide treatment strategies and help in monitoring resistance trends. AFST results were extracted from 45 studies (Figure 2) [25,26,27,28,29,30,31,32,33,34,35,36,37,38,39,40,41,42,43,44,45,46,47,48,49,50,51,52,53,54,55,56,57,58,59,60,61,62,63,64,65,66,67,68,69]. A cut-off was based on a North American study in 2023 that utilized a proposed resistance cut-off of minimum inhibitory concentration (MIC) ≥ 0.5 μg/mL [70]. For *T. rubrum*, a lesser degree of in vitro terbinafine resistance was observed. Clinical and Laboratory Standards Institute (CLSI) data revealed that over 80% of isolates had MIC_50_ values below 0.5 µg/mL. Similarly, per the European Committee on Antimicrobial Susceptibility Testing (EUCAST), more than 70% of isolates exhibited MIC_50_ values less than 0.5 µg/mL. When examining MIC_90_ results per CLSI, about 30% of *T. rubrum* isolates exhibited high MICs ≥ 0.5 µg/mL, while over 60% were below 0.5 µg/mL.

*T. indotineae* isolates exhibited a bimodal MIC_50_ distribution per the CLSI protocol, with approximately 30% of isolates showing MIC_50_ values greater than 0.5 µg/mL, while over 60% of isolates have MIC_50_ values below 0.5 µg/mL (Figure 2). The MIC_50_ distribution per EUCAST methodology reflected a similar bimodal pattern. However, when applying a different visual endpoint, more than 80% of *T. indotineae* isolates demonstrated MIC_90_ values equal to or greater than 0.5 µg/mL per CLSI. These findings indicate the need to standardize endpoint reading for growth inhibition to allow comparability between studies. In the absence of an established clinical breakpoint, AFST cannot reliably inform therapeutic decision making. Nonetheless, a resistance potential is clearly demonstrated by these in vitro observations.

### 3.3. Mechanisms of Resistance

**Figure 3:**
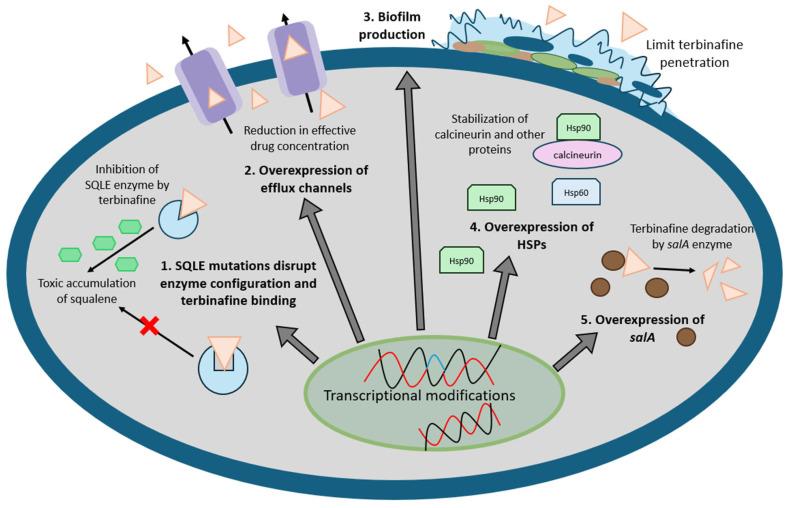
Mechanisms of terbinafine resistance in dermatophytes, encompassing disruption of terbinafine binding to squalene epoxidase (1), overexpression of efflux channel (2), biofilm production (3), heat shock proteins (4), and target degradation via the *salA* gene (5).

Antifungal resistance arises from innate resistance and adaptive changes due to prolonged antifungal pressure (Figure 3) [71]. Terbinafine works by disrupting the early stages of ergosterol biosynthesis by inhibiting the squalene epoxidase enzyme (SQLE) and preventing squalene oxidation [71]. This leads to the toxic accumulation of squalene and the depletion of ergosterol in the fungal cell membrane, disrupting membrane integrity and function, ultimately causing cell death [10,71,72]. Terbinafine resistance can be acquired in dermatophytes, which primarily stems from single nucleotide variations (SNVs) in the *SQLE* gene that alter enzyme conformation, reducing drug binding [10,73,74]. However, varying MIC values in isolates with identical *SQLE* mutations suggest additional mechanisms, including efflux pump overexpression, naphthalene degradation pathway activation, biofilm formation, and heat shock response upregulation [74,75].

#### 3.3.1. Single Nucleotide Variations (SNVs) in the Gene Encoding Squalene Epoxidase (SQLE)

Amino acid substitutions caused by SNVs in the *SQLE* gene result in structural changes that reduce terbinafine binding without disrupting the cell’s ergosterol biosynthesis pathway, resulting in resistance [75].

From our search, for *T. rubrum* isolates, Phe397Leu and Phe397Ile together comprise the most prevalent mutation (41.6% of isolates), followed by Leu393Phe (20.8%) and Ser395Pro (16.7%). Phe397Ile + Phe415Ser (8.3%) and Leu393Ser + Phe397Leu (4.2%) combination mutations are less common [29,36,37,45,51,61].

**Figure 4:**
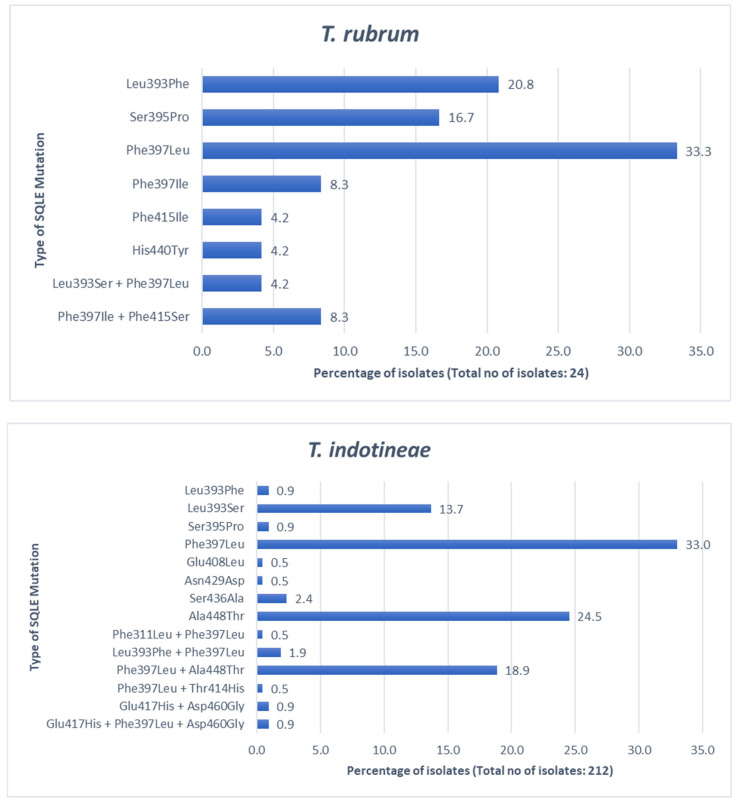
*SQLE* gene mutation distribution (in percentage) in *T. rubrum* (top panel, 24 isolates) vs. *T. indotineae* (bottom panel, 212 isolates) [28,29,30,31,34,36,37,39,42,44,45,50,51,52,55,61,62,66,76,77,78,79,80]. SNVs are indicated on the y-axis; isolate proportions are indicated on the x-axis.

In *T. indotineae*, Phe397Leu emerges as the most dominant (in 33.0% of isolates), followed by Ala448Thr (24.5%) and Phe397Leu + Ala448Thr combination mutation (18.9%). Leu393Ser occurs in 13.7% of isolates, followed by Ser436Ala (2.4%) [28,29,30,31,34,37,39,42,44,50,52,55,62,66,76,77,78,79,80]. His440Tyr was uniquely detected in *T. rubrum*, while Ala448Thr was unique to *T. indotineae* (Figure 4).

SNVs such as Phe397Leu, Leu393Phe, Leu393Ser, Phe415Ser, His440Tyr, and Phe484Tyr have been identified in terbinafine-resistant *Trichophyton* isolates [73,81,82,83]. The Ala488 position, also detected in terbinafine-susceptible *Trichophyton* isolates, is not directly adjacent to the terbinafine binding pocket, suggesting that Ala488 substitutions may not be crucial in terbinafine resistance development [30,34,81,83,84]. However, Ala448 has been associated with interfering ergosterol synthesis, potentially leading to decreased susceptibility to azole antifungals [83,85]. The single Ala448Thr substitution has been documented to be not a primary driver of terbinafine resistance on its own. While initial findings suggested a potential link to terbinafine resistance, subsequent evidence indicates that this mutation is found in both terbinafine-resistant and terbinafine-susceptible isolates [83]. Notably, this mutation is located on the surface of the SQLE protein, distant from the terbinafine binding pocket, which aligns with its lack of direct impact on terbinafine susceptibility [34]. In particular, cases of terbinafine resistance often involve both Ala448Thr and Phe397Leu mutations [83].

However, the presence of a single Ala448Thr mutation has been associated with increased MICs for azole antifungals [34,85,86]. In particular, De Paepe et al. (2024) identified five *T. indotineae* isolates: two with MIC_50_ values of 0.25 µg/mL for itraconazole, two with MIC_50_ values of 64 µg/mL for fluconazole, and one with MIC_50_ values of 0.25 µg/mL for voriconazole [31]. All these isolates contained the single Ala448Thr substitution [31].

The His440 position is reported to be closer to the binding pocket of terbinafine on the SQLE enzyme, and mutations have been associated with resistance in only some clinical isolates [82,83,87]. Single His440 substitutions have been associated with low-level terbinafine resistance in *Trichophyton* isolates. In one study, His440Tyr was found in *T. rubrum* isolates exhibiting low resistance [82].

#### 3.3.2. Drug Efflux Channels

Efflux membrane transporters, such as those belonging to the ATP binding cassette (ABC) superfamily or major facilitator superfamily (MFS), expel antifungal drugs from the cell [88,89]. Mutations in transcription factors can shift efflux pump expression from drug-inducible to constant, driving drug resistance [88,90,91].

Resistance is often linked to overexpression ABC transporter proteins, including pleiotropic drug resistance (PDR1) and multidrug resistance (MDR1, MDR2, and MDR4) [88,89,90]. Terbinafine exposure increases ABC-B transporter expression in *Trichophyton* strains, further supporting the role of efflux transporters in resistance [88]. *Microsporum canis* and *T. mentagrophytes* isolates have shown higher expression of *PDR1*, *MDR1*, *MDR2*, and *MDR4* [28,92]. Disrupting *MDR2* in a resistant *T. rubrum* strain enhances terbinafine susceptibility, underscoring the efflux transporter’s role in resistance [93]. Notably, some terbinafine-resistant isolates lacking *SQLE* mutations exhibit ABC transporter overexpression, suggesting efflux pumps alone can confer resistance [75,89,94].

#### 3.3.3. Biofilms

Dermatophyte biofilms are structured fungal communities embedded in an extracellular matrix, enabling adhesion to skin, nails, and medical devices [21,75]. Biofilms contribute to antifungal resistance, limiting drug penetration, forming structural barriers, and upregulating efflux pump genes [75,95]. While studied extensively in *Candida*, their role in dermatophyte resistance remains less explored [96]. *T. rubrum*, *M. canis*, and *T. mentagrophytes* can form biofilms in vitro exhibiting greater terbinafine resistance than planktonic cells [97,98,99,100]. Studies indicate that dermatophyte biofilms often require higher antifungal concentrations for effective inhibition or eradication [95].

#### 3.3.4. Heat Shock Proteins

Heat shock proteins (HSPs) act as molecular chaperones, produced in response to cellular stress to support cell survival and enhance fungal virulence by aiding host adaptation, promoting biofilm formation, and stabilizing proteins under stress, contributing to antifungal resistance [75,92,101]. Following terbinafine exposure, dermatophytes show increased expression of specific HSP genes, highlighting their role in the cellular stress response. Notably, in *T. rubrum*, terbinafine exposure upregulates multiple HSP genes, including *Hsp20*, *Hsp60*, *Hsp70*, and *Hsp90*, with terbinafine-resistant *T. indotineae* isolates showing higher baseline *Hsp60* and *Hsp90* expression levels [20,28,101].

Hsp90 aids antifungal resistance by stabilizing calcineurin, which activates pathways necessary for resistance [102,103,104]. Crz1, a key downstream effector, links calcineurin activation to azole resistance in *Candida albicans* (*C. albicans*) [91,92,105]. Dephosphorylated Crz1 regulates genes involved in signaling, ion transport, cell wall integrity, and vesicular trafficking [91,103,104]. Deleting the CNA-1 gene in *C. albicans* increases terbinafine sensitivity [103]. While the interactions between HSPs, calcineurin, and terbinafine are not fully understood, calcineurin likely induces genetic or cellular changes that help fungi adapt to stress [103,104,106]. Though much research has focused on *C. albicans*, the calcineurin pathway is conserved in virulence and pathogenicity [104]. Since calcineurin regulates ergosterol biosynthesis, antifungals like terbinafine and azoles may share similar resistance mechanisms involving this pathway.

#### 3.3.5. Naphthalene Degradation

The *salA* gene, which encodes salicylate 1-monooxygenase in the naphthalene degradation pathway, has been proposed as a terbinafine resistance mechanism. Since terbinafine contains a naphthalene ring, it may serve as a *salA* substrate, leading to drug degradation [107,108,109]. The presence of terbinafine has been proposed to promote the accumulation of *salA* transcripts and overexpression of this gene, potentially due to a multicopy effect, potentiating resistance to terbinafine [107]. In *T. rubrum* isolates, challenging with cytotoxic drugs, including terbinafine, has been shown to cause overexpression of the *salA* gene [107,110]. Moreover, plasmids containing the *salA* gene were used to successfully transform a *T. rubrum* strain from terbinafine-sensitive to terbinafine-resistant. This suggests that a similar resistance mechanism may enable *T. rubrum* to counteract the inhibitory effects of terbinafine [109].

## 4. Treatments for Terbinafine Resistance

*T. indotineae* exhibits higher antifungal resistance compared to *T. rubrum* [70]. Current treatments for *T. rubrum* and *T. indotineae* primarily involve terbinafine and the azoles. Typically, topical therapies are preferred in milder cases, while oral antifungals are for widespread infections. In severe cases, combination therapy, which integrates both the topical and oral approach, may be considered (Table 2) [111]. Treatment strategies combining agents with different modes of action can reduce the likelihood of resistance development; for instance, topical ciclopirox—a broad-acting antifungal—has been tried in combination with terbinafine treatment [112,113,114]. In cases of terbinafine-resistant *T. indotineae* infection, combinations of oral itraconazole with topical azoles are commonly utilized to help ensure a sufficient dosage is reached at the site of infection. The latter includes topical azoles such as bifonazole, clotrimazole, ketoconazole, luliconazole and sertaconazole [86,115,116,117,118,119].

Azoles and terbinafine interfere with fungal ergosterol synthesis. Itraconazole, which needs to be taken with food, inhibits lanosterol 14α-demethylase, and terbinafine inhibits squalene epoxidase [120]. Griseofulvin inhibits dermatophyte growth by disrupting fungal microtubules, impairing mitosis, with a half-life of 9–24 h [121]. The effective half-lives of terbinafine and itraconazole are ~36 h and 16–24 h in adults, respectively [122].

In the case of glabrous infections, oral terbinafine is dosed at 250 mg/day for 4–8 weeks for *T. rubrum* and 4–12 weeks for *T. indotineae* [111]. Itraconazole and the super-bioavailable variant (SUBA-itraconazole) are generally effective against *T. rubrum* (itraconazole—200 mg daily for 4–8 weeks; SUBA-itraconazole—130 mg daily for 4–6 weeks) and *T. indotineae* (itraconazole—200 to 400 mg daily for 4–12 weeks; SUBA-itraconazole—130 mg daily for 6–8 weeks) [111].

Oral voriconazole and posaconazole are also effective against both *T. rubrum* and *T. indotineae*; however, they are typically reserved for resistant cases due to side-effects [70,123]. Fluconazole is less effective than terbinafine and itraconazole against *T. rubrum*, and griseofulvin may be effective against *T. rubrum* only with prolonged treatment [111,124]. Fluconazole and griseofulvin are not recommended for *T. indotineae* due to reported high-level resistance [70,124]. Adverse effects of oral antifungals can include gastrointestinal disturbances and hepatotoxicity; with itraconazole caution needs to be exercised with cardiovascular risks [125].

Topical terbinafine and voriconazole creams are available for localized resistant infections. Local adverse effects include burning irritation, erythema, and dermatitis [125]. Typical dosing for voriconazole cream is 1% compounded and applied 1–2 times a day for 1–4 weeks; topical terbinafine is dosed 2–3 times daily for 2–6 weeks [123].

**Table 2:** Summary of Antifungal Treatments for *T. rubrum* vs. *T. indotineae*.

Drug	Mechanism	Route	Pulse Therapy	Continuous Therapy	*T. rubrum*	*T. indotineae*
Terbinafine	Inhibits squalene epoxidase	Oral, topical	250 mg BID, 1 week/month (not commonly used)	250–500 mg/day, 4–12 weeks	Highly effective [124]	High resistance [124]
Griseofulvin	Inhibits microtubule function	Oral	Rarely used in pulse	10–20 mg/kg/day, 6–12 weeks	Somewhat effective but therapy for long duration [21]	Limited data, uncertain efficacy [21]
Itraconazole	Inhibits lanosterol 14α-demethylase	Oral	200 mg BID, 1 week/month, 1–3 pulses (superficial fungal infections); 3–4 or more pulses (onychomycosis)	100–200–400 mg/day, 4–12 weeks	Effective [126]	Preferred alternative to terbinafine [126]
Voriconazole	Inhibits lanosterol 14α-demethylase	Oral, topical	Not typically used	200–400 mg/day	Effective but rarely needed [111]	Effective in resistant cases. Topical voriconazole effective in selected cases [111]
Posaconazole	Inhibits lanosterol 14α-demethylase	Oral	Not commonly used	300 mg/day	Effective but rarely needed [127]	Used in recalcitrant cases [70]
Fluconazole	Inhibits lanosterol 14α-demethylase	Oral	150–300 mg once weekly	100–200 mg/day	Less effective than terbinafine/itraconazole [21]	Low efficacy, resistance reported [21]

## 5. Antifungal Stewardship

**Figure 5:**
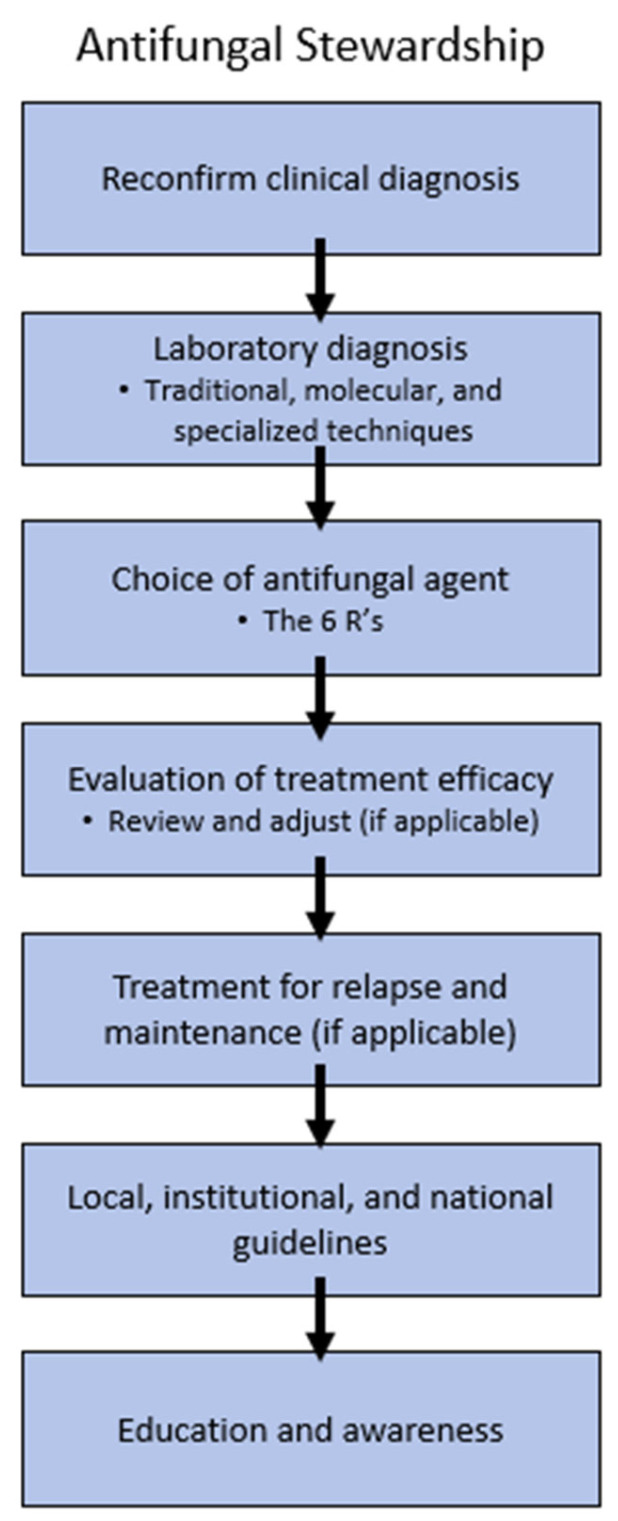
Critical Strategies for Antifungal Stewardship.

Antifungal stewardship (AFS) is a strategy focused on optimizing antifungal use to improve patient outcomes, minimize resistance, and reduce toxicity [2,128,129]. By promoting appropriate prescribing practices and surveillance, AFS ensures effective treatment while safeguarding the current selection of antifungal therapies against growing resistance threats (Figure 5).

### 5.1. Reconfirm Clinical and Laboratory Diagnosis

Misuse of antifungals and misdiagnosis of fungal infections can worsen outcomes, drive resistance, and delay proper treatment [11]. Laboratory testing, comprising traditional (KOH microscopy and culture), molecular (PCR), and specialized (antifungal susceptibility testing, *SQLE* mutation identification, sequencing) methods, should complement clinical assessment to confirm diagnoses and guide treatment. Despite diagnostic limitations, prioritizing microbiologic confirmation over empiric treatment is crucial for optimizing antifungal therapy and preventing resistance [129].

### 5.2. Choice of Antifungal Agent

The selection of an appropriate antifungal agent to treat dermatophyte infections can be understood using six principles, referred to as the six Rs: right drug, right indication, right route of administration, right dose, right duration of treatment, and right frequency [2]. Optimal antifungal selection depends on fungal species, infection site, pharmacokinetics, and patient-specific factors like comorbidities and drug interactions.

### 5.3. AFST

AFST can inform on antifungal drug efficacy in vitro; however, in vitro resistance does not always correlate with clinical resistance [10,24]. Continued efforts in conducting AFST studies can help better inform future protocol amendments and standardization, as well as increasing its clinical utility [2,130].

Managing treatment relapse and maintenance therapy is key to preventing recurrent fungal infections and improving long-term outcomes. Relapse may result from incomplete pathogen eradication, poor adherence to the treatment protocol, or underlying co-morbid conditions. In cases of relapse, AFST can guide adjustments to antifungal therapy [11].

Maintenance therapy is necessary for patients with chronic or recurrent infections, particularly those with immunosuppression or persistent risk factors [131,132,133]. Long-term strategies should balance efficacy with minimizing toxicity and resistance, emphasizing patient education on adherence and regular follow-up to ensure continued success.

### 5.4. Institutional, Local, and National Guidelines

The WHO’s 2015 global action plan and 2022 priority pathogen list emphasize the need for continued research, especially in low-resource settings, to enhance treatment, diagnostics, and resistance strategies [134,135]. Effective AFS requires a multidisciplinary approach, with dermatologists, pharmacists, and microbiologists working together to optimize treatment, minimize prolonged therapy, and guide de-escalation [2,128,136,137]. Given regional resistance differences, a flexible, collaborative AFS program based on the resources and needs of the regional patients can inform diagnosis and treatment decisions and improve patient outcomes and institutional resistance monitoring [129,136,137].

### 5.5. Education and Awareness

A comprehensive approach includes workshops and social media to increase health care professionals’ understanding of the importance of antifungal stewardship and recognition of signs of resistance is recommended [8]. Public health campaigns focused on educating health care professionals to identify dermatophyte infections and educating patients on the importance of completing prescribed regimens and avoiding self-medication can help reduce the impact and spread of antifungal-resistant dermatophytes [2,138].

Disinfection of laundry helps curb the dermatophyte infection load [139]. Dermatophyte spores, particularly arthrospores, can remain present on textiles and surfaces for extended periods [140,141]. Moderate heat treatments can destroy most dermatophytes and their spores (e.g., laundering socks at temperatures of 60 °C or higher) [139,140,141]. Alternatively, 24 h soaking of contaminated textiles in a quaternary ammonium compound (QAC) detergent can achieve complete disinfection [140]. Notably, textile and attire types influence disinfection efficacy. For example, cotton is susceptible to fungal contamination due to high moisture absorption; hence, higher-temperature laundering is recommended [142]. For shoes, ozone sanitization can be considered [139,141,143]. Regular disinfection of more susceptible surfaces, such as bathroom floors, with 10% bleach solutions is recommended to eliminate fungal spores and prevent persistence [144].

## Figures and Tables

**Table 1:** Clinical characteristics of *T. rubrum* and *T. indotineae* infections.

	*Trichophyton rubrum*	*Trichophyton indotineae*
Summary	Currently the most prominent dermatophyte worldwide. Distinguishing morphological features in KOH and culture	Terbinafine-resistant *Trichophyton mentagrophytes* with ITS genotype VIII May be indistinguishable from *Trichophyton mentagrophytes/interdigitale* complex unless sequenced
Common lesion sites	Tinea pedis Tinea corporis Tinea cruris Onychomycosis	Tinea corporis Tinea cruris Tinea faciei
Lesion characteristics	Annular, scaly plaques with central clearing Dry scaling on soles: tinea pedis Chronic, mild progression Common nail infection: thickened, discolored, brittle nails Scalp involvement uncommon	Intense pruritus Erythematous, scaly plaques Multiple concentric ring-on-ring patterns Severe, chronic, and recalcitrant lesions Nail involvement uncommon Scalp involvement uncommon
Response to terbinafine	Generally, responds to terbinafine, although squalene epoxidase (*SQLE*) mutations have been reported in resistant cases	Highly resistant to terbinafine Exhibit squalene epoxidase (*SQLE*) mutations and resistance to terbinafine Itraconazole or combination therapies (oral plus topical) may be required

## Data Availability

No new data were created or analyzed in this study.
